# Dotted Pigmentation of the Cuticle

**DOI:** 10.1111/pde.70139

**Published:** 2026-02-09

**Authors:** Anna Bolzon, Antonella Tosti

**Affiliations:** ^1^ Dermatology Unit, Department of Medicine (DIMED) University of Padua Padua Italy; ^2^ Dr Phillip Frost Department of Dermatology and Cutaneous Surgery University of Miami School of Medicine Miami Florida USA

**Keywords:** cuticle pigmentation, Hutchinson sign, melanin granules, melanonychia, nail matrix nevus, pediatric dermoscopy, pseudo‐Hutchinson sign

## Abstract

Longitudinal melanonychia in children is usually caused by nail matrix nevi and often fades over time. Pediatric patients with this finding are frequently referred to dermatologists due to concerns that it may represent melanoma. We report a case of a 4‐year‐old girl with longitudinal melanonychia on the left second fingernail, accompanied by pigmented dots in the cuticle that persisted after the melanonychia had faded.

To the Editors,

A 2‐year‐old girl presented with a band of longitudinal melanonychia on the left second fingernail, first noted by the parents at the age of 6 months. The band involved approximately 80% of the nail plate and showed an irregular pattern over a brown background, with longitudinal lines irregular in color, width, and thickness, as well as pigmented dots visible along the lines on dermoscopy (Figure 1A). A nail matrix biopsy revealed a nail matrix nevus.

**FIGURE 1:**
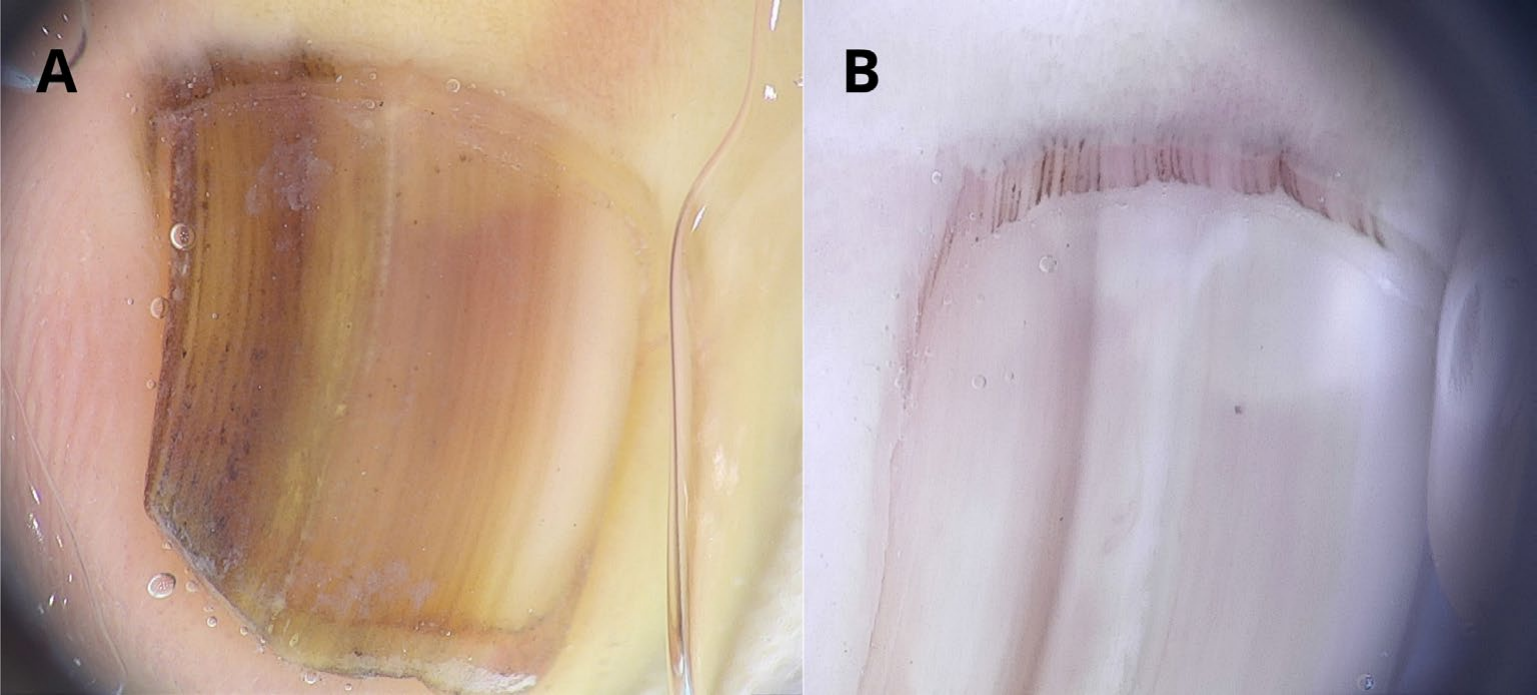
(A) Onychoscopy of the longitudinal melanonychia with irregular pigmented bands of variable thickness, containing pigmented dots. (B) At follow‐up: Longitudinal melanonychia with pale brown bands of variable thickness. Pigmented dots arranged in parallel bands within the cuticle.

During follow‐up, the band gradually faded. At 4 years of age, the band involved less than 50% of the nail plate and appeared pale brown with lines irregular in color and thickness. Pigmented dots arranged in linear parallel bands were present within the cuticle, both in correspondence with and at the periphery of the band, where the nail plate pigmentation had faded (Figure 1B).

Longitudinal brown‐to‐black bands in the nail plate are due to the presence of melanin within the nail plate. In pediatric patients, they primarily correspond to nail matrix nevi, while nail matrix melanoma is extremely rare in this population [1].

Fading and complete regression of nail matrix nevi in pediatric patients have been reported in the literature [1, 2]. It is well established that, in children, fading of nail pigmentation is due to a reduction in melanin production by the nevus cells rather than to regression of the nevus itself [2]. Moreover, the presence of pigmented dots distributed along melanotic lines in the nail plate has been described as a dermoscopic feature of benign regression in cases of longitudinal melanonychia in the pediatric population [3]. In contrast, in adults the presence of pigmented dots along melanocytic lines on onychoscopy is a highly suspicious sign of nail matrix melanoma [4].

Dotted pigmentation of the cuticle must be distinguished from the pseudo‐Hutchinson sign and the Hutchinson sign. The pseudo‐Hutchinson sign in children is benign and consists of melanin pigment on the nail plate that is visible through the transparent cuticle and the thin free margin of the proximal nail fold, without extension of pigment into the surrounding skin (Figure 2A). In contrast, the Hutchinson sign corresponds to melanin pigment extension into the periungual skin (proximal or lateral nail fold, or hyponychium) [5] (Figure 2B), and may represent malignancy [5].

**FIGURE 2:**
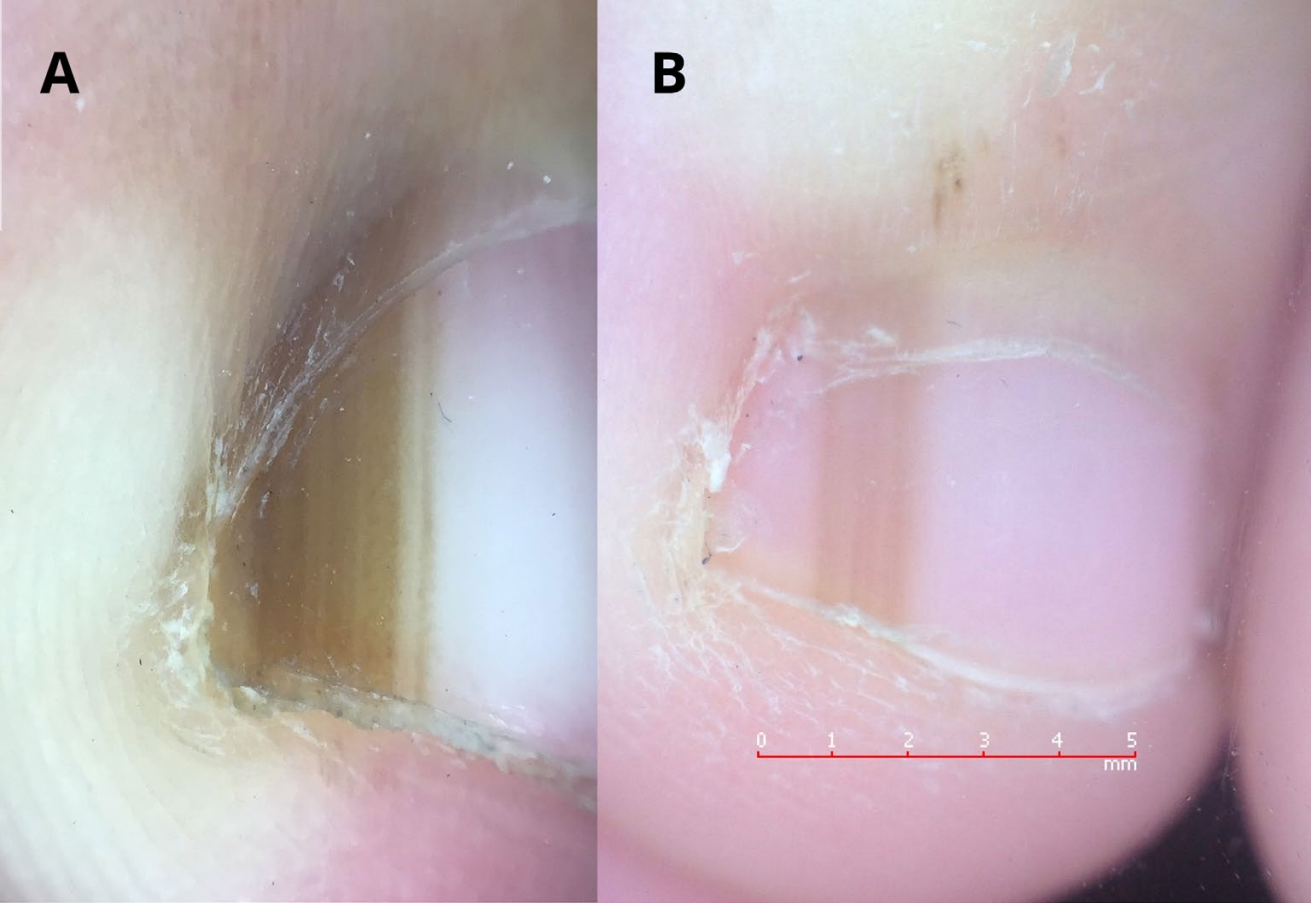
(A) Hutchinson sign: Pigmentation of the periungual tissue. (B) Pseudo‐Hutchinson sign: Nail plate pigmentation visible through the cuticle and thin free margin of the proximal nail fold.

In our case, pigmentation appeared as discrete dots aligned in linear bands within the cuticle, without continuous brown discoloration of the periungual skin or pigment visible beneath the transparent nail fold. As previously reported by Maddy et al., we speculate that the dotted pigmentation of the cuticle is due to the presence of quiescent melanocytic nests located in the proximal nail matrix and in the ventral portion of the proximal nail fold, which produces the cuticle. Therefore, this finding should be considered benign and should not be mistaken for either the pseudo‐Hutchinson or the Hutchinson sign.

## Author Contributions

A.T. and A.B. conceived the idea. A.B. contributed to the writing of the manuscript. A.T. helped with revision and supervision.

## Conflicts of Interest

The authors declare no conflicts of interest.

## Data Availability

The data that support the findings of this study are available from the corresponding author upon reasonable request.
